# Enhancing nasopharyngeal carcinoma cell radiosensitivity by suppressing AKT/mTOR via CENP-N knockdown

**DOI:** 10.1186/s12967-023-04654-x

**Published:** 2023-11-08

**Authors:** Li-Zhi Wu, You Zou, Bin-Ru Wang, Hai-Feng Ni, Yong-Gang Kong, Qing-Quan Hua, Shi-Ming Chen

**Affiliations:** 1 Department of Otolaryngology-Head and Neck Surgery, Renmin Hospital of Wuhan University, 238 Jie-Fang Road, Wuhan, 430060 Hubei People’s Republic of China; 2 Department of Otolaryngology Head and Neck surgery, Affiliated Hangzhou First People’s Hospital, Zhejiang University School of Medicine, Hangzhou, 310006 Zhejiang People’s Republic of China; 3 Institute of Otolaryngology-Head and Neck Surgery, Renmin Hospital of Wuhan University, 238 Jie-Fang Road, Wuhan, 430060 Hubei People’s Republic of China

**Keywords:** Nasopharyngeal carcinoma, CENP-N, Radiosensitivity, Apoptosis, Cell cycle, AKT/mTOR signaling pathway

## Abstract

**Objective:**

Investigating the impact of centromere protein N (CENP-N) on radiosensitivity of nasopharyngeal carcinoma (NPC) cells.

**Methods:**

Using immunohistochemistry and immunofluorescence to detect CENP-N expression in tissues from 35 patients with radiosensitive or radioresistant NPC. Assessing the effect of combined CENP-N knockdown and radiotherapy on various cellular processes by CCK-8, colony formation, flow cytometry, and Western blotting. Establishing a NPC xenograft model. When the tumor volume reached 100 mm^3^, a irradiation dose of 6 Gy was given, and the effects of the combined treatment were evaluated in vivo using immunofluorescence and Western blotting techniques.

**Results:**

The level of CENP-N was significantly reduced in radiosensitive tissues of NPC (p < 0.05). Knockdown of CENP-N enhanced NPC radiosensitivity, resulting in sensitizing enhancement ratios (SER) of 1.44 (5-8 F) and 1.16 (CNE-2Z). The combined treatment showed significantly higher levels of proliferation suppression, apoptosis, and G2/M phase arrest (p < 0.01) compared to either CENP-N knockdown alone or radiotherapy alone. The combined treatment group showed the highest increase in Bax and γH2AX protein levels, whereas the protein Cyclin D1 exhibited the greatest decrease (p < 0.01). However, the above changes were reversed after treatment with AKT activator SC79. In vivo, the mean volume and weight of tumors in the radiotherapy group were 182 ± 54 mm^3^ and 0.16 ± 0.03 g. The mean tumor volume and weight in the combined treatment group were 84 ± 42 mm^3^ and 0.04 ± 0.01 g.

**Conclusion:**

Knockdown of CENP-N can enhance NPC radiosensitivity by inhibiting AKT/mTOR.

**Supplementary Information:**

The online version contains supplementary material available at 10.1186/s12967-023-04654-x.

## Introduction

Nasopharyngeal carcinoma (NPC) is not a common tumor on a global scale. Statistics show that new cases of NPC accounted for only 0.7% of all newly diagnosed cancer patients in 2018 [1]. Furthermore, its geographical distribution is extremely uneven. Specifically, the age-standardized incidence rates (ASIRs) of NPC is less than 1/100,000 in the majority of regions worldwide. However, in southern China, ASIRs of NPC is notably higher, reaching 20/100,000. In addition, the incidence rate remained high after the population migrated from high-risk areas to non-endemic areas, and it did not begin to decline until the second generation of immigrants [2]. Significant racial and geographical distribution differences of NPC and the common Epstein-Barr virus (EBV) infection among the affected population suggest that the incidence of nasopharyngeal cancer is influenced by various factors, such as viral infection, predisposition, and environmental conditions [3]. Due to the radiosensitivity and deep anatomical location of NPC, radiotherapy is the main treatment for nonmetastatic NPC. However, we reviewed literatures on NCBI about the therapeutic effect of NPC in the past 20 years, and found that 20% of patients experience local recurrence and distant metastasis after radiotherapy, primarily due to the development of radiotherapy resistance [4].

Changes in tumor microenvironment, gene mutation, abnormal gene expression, gene epigenetic modification, or abnormal activation of some signaling pathways are the main causes of radiotherapy resistance in nasopharyngeal carcinoma [5]. Gene targeted therapy can specifically target the abnormally expressed genes in radioresistant nasopharyngeal carcinoma and reduce the occurrence of radioresistance. Compared with other treatment methods, it is more accurate and can reduce the radiation dose of NPC, thereby reducing the damage of radiotherapy to surrounding healthy tissues [6]. Chen et al. found that targeting the USP44-TRIM25-Ku80 signaling axis can enhance the radiosensitivity of nasopharyngeal carcinoma [7]. According to a reseatch conducted in 2014, SHP-1 (protein tyrosine phosphatase 1 containing SH2 structure) can up-regulate the production of Cyclin D1, hence increasing the cycle of nasopharyngeal cancer cells. There is a quickening in the pace at which cells replicate. As a result, the SHP-1 gene has the ability to modulate the sensitivity of cells via controlling the production of the cyclin D1 gene. In the case of nasopharyngeal cancer cells, suppressing the SHP-1 gene through the use of shRNA technology can cause the cells cycle to slow down and enter the S phase, which in turn increases their sensitivity to radiotherapy [8]. Di et al. found that deubiquitination of OTUD4 and stabilization of GSDME could enhance radiation-induced pyroptosis, thereby increasing the radiosensitivity of NPC [9]. Therefore, it is expected to significantly improve the survival and prognosis of nasopharyngeal carcinoma patients by exploring the key genes of radiotherapy resistance and targeting them.

AKT is a serine/threonine kinase that participates in multiple vital cellular processes, such as angiogenesis, invasion, proliferation, etc. [10]. mTOR, by interacting with different protein molecules, forms two complexes substances: mTORC1 and mTORC2. mTORC1 regulates cellular glucose metabolism and lipid synthesis by activating downstream effectors, such as p70S6K and 4EBP1. mTORC2 activates PKC-α and AKT, regulating the actin cytoskeleton in cells [11].

According to recent studies, the activation of AKT/mTOR signaling pathway leads to radiotherapy resistance in various cancers, and many valuable inhibitors have been developed targeting AKT/mTOR, such as MK-2206, AZD5363, BI860585, GDC-0349, etc. [12]. Studies have found that MK-2206 can increase DNA double-strand breaks, decrease cell cycle checkpoint inactivation and DNA repair protein expression, and enhance the radiation sensitivity of glioma cells [13]. However, since the AKT signaling pathway intersects with many other signaling pathways, AKT inhibitors are prone to off-target effects. For example, in some clinical trials, it was found that transient hyperglycemia and some toxic effects, including immunosuppression, cardiotoxicity, neuropsychiatric effects, hepatotoxicity, etc., were prone to occur after the use of the allosteric AKT inhibitor MK2206 [14]. In addition, rapamycin and its analogs have been developed as mTOR inhibitors for cancer therapy. However, rapamycin causes feedback activation of AKT [12]. In-depth exploration of off-target and side effects mechanisms of AKT/mTOR inhibitors may help overcome radiotherapy-resistant tumors more safely and effectively.

Centromere protein N (CENP-N), located on chromosome 16q23.2, also called BM039, ICEN32, or C16orf60, plays a critical role in centromere and kinetochore stability by directly interacting with CENP-A during early mitosis and recruiting CENP-L to form the CENP-L/N complex [15]. When extraterrestrial factors, such as radiation, induce DNA double-strand breaks, specific proteins, including CENP-A and CENP-N, are recruited to repair the damage at the break. The absence of CENP-N may inhibit the repair of DNA damage, which will initiate programmed cell death [16]. CENP-N is overexpressed in various cancers, such as oral squamous cell carcinoma, esophageal cancer, liver cancer, etc. Moreover, high CENPN expression is often closely associated with poor survival outcomes [17–19]. Wu et al. identified high expression of CENP-N as a novel biomarker for adverse prognosis in 1833 patients with glioblastoma using bioinformatics analysis of The Cancer Genome Atlas (TCGA) [20]. Nevertheless, the correlation between the expression of CENP-N and radiosensitivity in NPC is still not well understood.

**Scheme 1:**
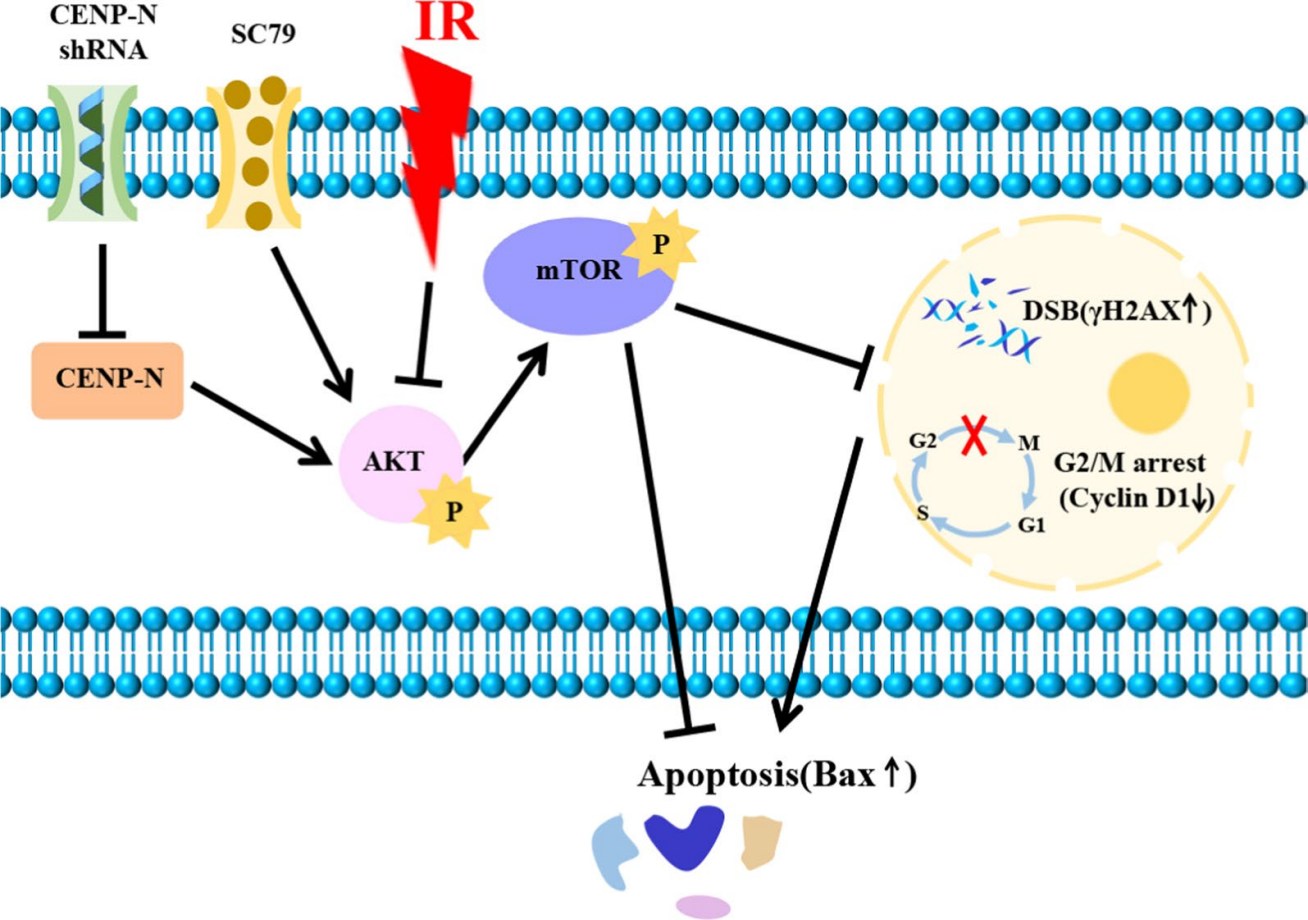
Enhancing nasopharyngeal carcinoma cell radiosensitivity by suppressing AKT/mTOR via CENP-N knockdown.

In this study, short hairpin RNA targeting CENP-N was employed to examine the impact of CENP-N on the modulation of NPC’s radiosensitivity and to explore its molecular mechanism (Scheme 1).

## Materials and methods

### Human tissue samples

A retrospective analysis was conducted using biopsy samples collected from 35 patients with nonkeratinizing nasopharyngeal squamous cell carcinoma in the Department of Pathology, Renmin Hospital of Wuhan University from May 2020 to December 2021. The biopsy samples were fixed with formaldehyde for fixation and subsequently transformed into paraffin blocks for further examination. The assessment of the therapeutic response was conducted in accordance with the RECIST 1.1 standard [21]. The radiosensitive group consisted of 20 patients who achieved complete or partial remission, while the radioresistant group included 15 patients with stable or progressive disease. Approval for this study was granted by the Ethics Committee of Renmin Hospital of Wuhan University, with the assigned approval number 2020 K-K221(Y01).

### Immunofluorescence analysis

The tissue sections were dewaxed and hydrated with gradient ethanol. After antigen repair, they were subsequently subjected to treatment with a 10% solution of bovine serum albumin (BSA, Yamei, Shanghai, China) for 60 min. Subsequently, incubated with the primary antibody (1:800) overnight. Subsequently, they were incubated with fluorescent secondary antibodies (1:500) without light. Then nuclei were stained with DAPI (5 µg/mL). ImageJ was used to evaluate integrated optical density (IOD) (IOD = area × intensity of fluorescence) [22]. Additional file 3: Table S1 described the information of antibodies.

### Immunohistochemical analysis

The previous steps are the same as for immunofluorescence except staining with diaminobenzidine. The staining index is calculated by multiplying the staining intensity (0 for negative, 1 for light yellow, 2 for medium yellow, and 3 for intense brown) with the positive cell ratio (1 for less than 10%, 2 for 10–50%, and 3 for more than 50%). In this study, we defined the staining index of 0–3 as CENP-N low expression and 4–9 as CENP-N high expression [23].

### Cell culture and transfection

The NPC cell lines 5-8 F and CNE-2Z derived from human were donated by Southern Medical University of China and cultured in RPMI 1640 medium (Servicebio, Wuhan, China), containing 10% FBS (Biological Industries, Israel) at 37 °C, 5% CO_2_.

Lipofectamine transfection reagent, shCENP-N plasmid and packaging plasmids (pMD2G and psPAX2) were mixed to transfect 293T. After 48 hours, collecting the lentivirus-containing supernatant to infect NPC cells. Then purinomycin was used for one week to kill cells that were not successfully transfected. The CENP-N shRNA sequence is 5′-GCCCTGTTAGACATCATTCAAGAGATGATGTCTAACAGGGC-3′.

### Cell irradiation

The cells were exposed to vertical irradiation using an X-ray Varian linear accelerator at a rate of 400 cGy per minute for a duration of 1 min.

### CCK-8 assay

In each well, 5000 cells were seeded and incubated for 24, 48, and 72 h. Afterward, 10% CCK8 reagent (Dojindo, Shanghai, China) was added and the cells were incubated for an additional hour. The absorbance value at 450 nm was detected with a microplate reader (Perkin-Elmer, MA, USA).

### Colony formation assay

In each well, 1000 cells were seeded and subjected to radiation doses of 2, 4, or 6 Gy. Following a period of 10 days, the samples were treated with methanol and then subjected to staining using 1% crystal violet. The survival fraction (SF) = colony number/seeded cells × 100%. The cell survival curve was fitted using Prism8 software [24].

### Cell cycle assay

A total of 1 × 10^6^ cells were incubated with 500 µL cell cycle staining solution and 10 µL PI at 37 °C for 30 min without light. A CytoFLEX S flow cytometer (Beckman, USA) was used to detect the cell cycle distribution.

### Apoptosis assay

Annexin V-APC and 7-AAD were used to stain a grand total of 1 × 10^6^ cells (Beyotime, Shandong, China). A CytoFLEX S flow cytometer (Beckman, USA) was used to detect apoptotic cells, and the gating strategy was shown in Additional file 2: Fig. S2B.

### Western blot analysis

The proteins were separated by 10% SDS-PAGE gel (Servicebio, Wuhan, China) and then transferred to PVDF membranes (Millipore, MA, USA). After incubation with a protein-free rapid sealing (EpiZyme, Shanghai, China) for 10 min, the membranes were then incubated overnight at 4 °C with antibodies (1:800). Then, the membranes were treated with a goat anti-rabbit secondary antibody (1:3000) for 1 h. Using a chemiluminescence reagent (Beyotime, Shandong, China) to visualize the proteins. Grayscale values were measured by image lab, with β-actin serving as the internal control.

### Xenograft radiotherapy model

A total of 24 female BALB/c nude mice, aged four weeks, were raised in a specific pathogen-free (SPF) environment at the Animal Experimental Center of Renmin Hospital of Wuhan University, these mice were then randomly assigned to four groups, 5 × 10^6^ cells were injected subcutaneously into the right leg of each mouse. Starting from the 6th day, tumor dimensions (length and width) were measured every other day. Once the tumor volume reached 100 mm^3^, the local irradiation dose was 8 Gy at 600 cGy/min for 1 min. On the 16th day after radiotherapy, the nude mice were euthanized, and the xenograft tumors were collected. Approval for this animal experiment was granted by the Animal Ethics Committee of Renmin Hospital of Wuhan University (Approval No.: WDRM20200815).

### Statistical analysis

All experiments were biologically repeated three times. Prism8 statistical software (GraphPad, USA) was utilized for the analysis of the experimental data. The mean ± standard deviation (mean ± SD) is used to present quantitative data. Group differences were assessed using one-way analysis of variance (ANOVA) followed by an LSD-t test and post-hoc Bonferroni’s correction for multiple comparisons. p<0.05 was deemed to be statistically significant. For every experiment, when the value of p is less than 0.05, it is denoted as *; when it is less than 0.01, it is denoted as **; and when it is less than 0.001, it is denoted as ***.

## Results

### CENP-N is significantly reduced in radiosensitive NPC

**Fig. 1:**
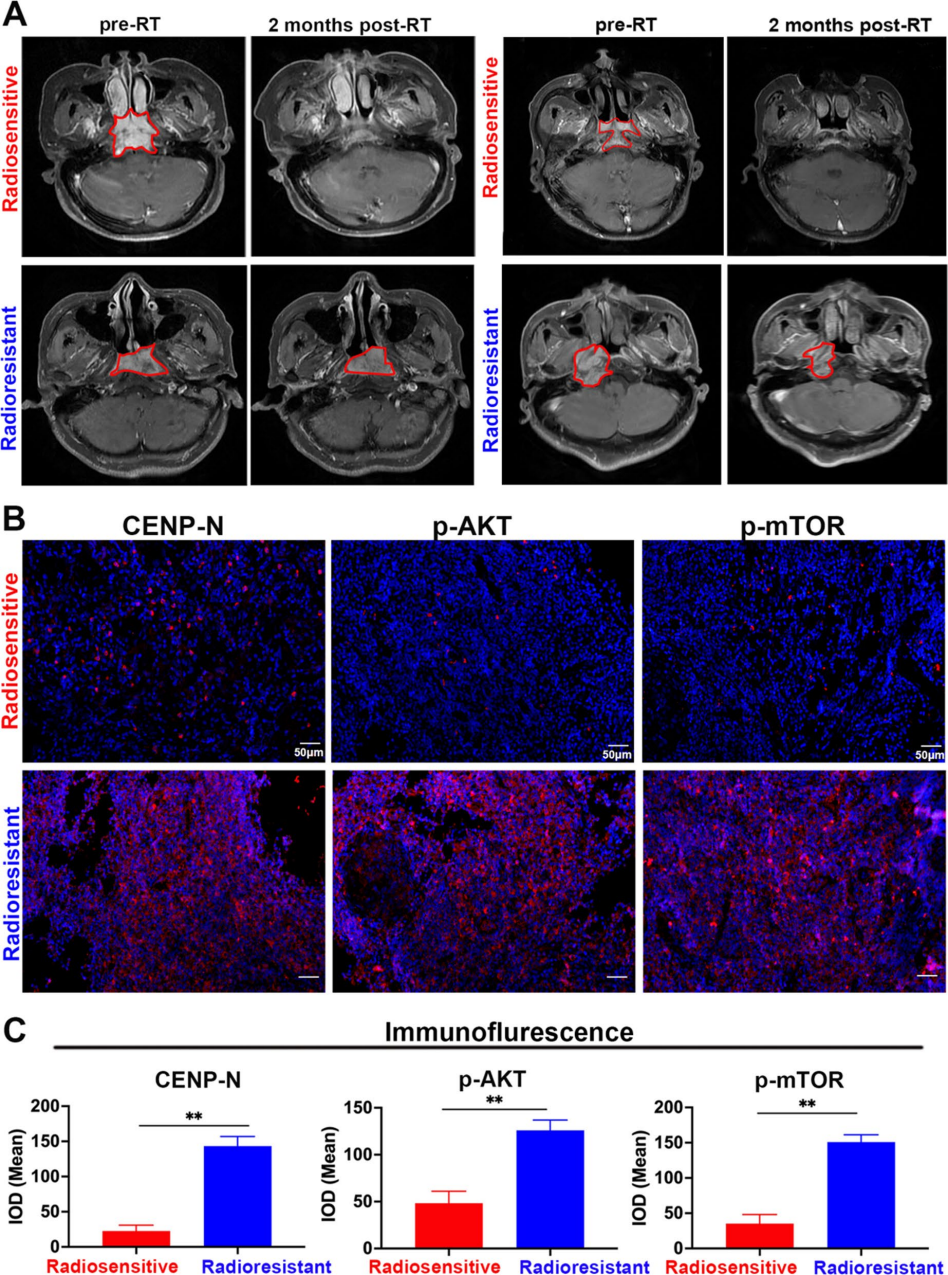
CENP-N is significantly reduced in radiosensitive NPC. **A** Representative MRI of radiotherapy-sensitive and radiotherapy-resistant NPC patients, with the red line indicating tumor tissue. **B** Representative immunofluorescence images of CENP-N, p-AKT, and p-mTOR in 35 radiotherapy-sensitive and radiotherapy-resistant tissue samples. **C** Immunofluorescence staining intensity of CENP-N, p-AKT, and p-mTOR in 35 NPC samples, data were analyzed using Student’s *t*-test, **p < 0.01

Firstly, we collected 20 radiosensitive NPC tissue samples and 15 radioresistant NPC tissue samples. Figure 1A shows representative magnetic resonance images (MRI) of patients with radiosensitive and radioresistant NPC, the radiologist outlined the scope of nasopharyngeal cancer with a red line. Radiosensitive patients showed significant tumor regression after radiotherapy, while radioresistant patients showed slow tumor regression after radiotherapy. We analyzed the expression of CENP-N, p-AKT, and p-mTOR in 35 NPC tissue samples and found that compared with those in radioresistant tissue samples, CENP-N, p-AKT, and p-mTOR expression in radiosensitive samples were significantly reduced (Fig. 1B, C).

Table 1 summarizes the general characteristics of 35 NPC patients. We found no correlation between CENP-N expression and age or sex. However, high CENP-N expression is closely related to radioresistance of NPC (p < 0.01). CENP-N expressions in the radiosensitive and radioresistant NPC samples were scored by immunohistochemical staining. Representative immunohistochemical images were shown in Additional file 1: Fig. S1A. In current classification of malignant tumors, NPC still belongs to the subtype of head and neck squamous cell carcinoma (HNSCC), and there is no separate classification of NPC in any database. Therefore, we used GEPIA2 database [25–27] to analyze CENP-N expression in HNSCC and para-canceous tissues, As shown in Additional file 1: Fig. S1C, a significant upregulation of CENP-N in HNSCC was observed when compared to para-canceous tissues.

**Table 1:** Clinical characteristics of the patients with NPC stratified by CENP-N expression

	CENP-N expression			
	Case	High	Low	P value
Sex				0.5051
Male	18	10(55.6%)	8(44.4%)	
Female	17	7(41.2%)	10(58.8%)	
Age, years				0.3670
≥ 50	29	11(37.9%)	18(62.1%)	
<50	6	4(66.7%)	2(33.3%)	
Treatment				
No	35	17	18	
Yes	0	0	0	
Groups				0.0012**
Radiotherapy-resistant	15	11(73.3%)	4(26.7%)	
Radiotherapy-sensitive	20	3(15%)	17(85%)	

P values were calculated using the chi-square test; **, p < 0.01.

### Knockdown of CENP-N inhibits NPC proliferation and enhances radiosensitivity

**Fig. 2:**
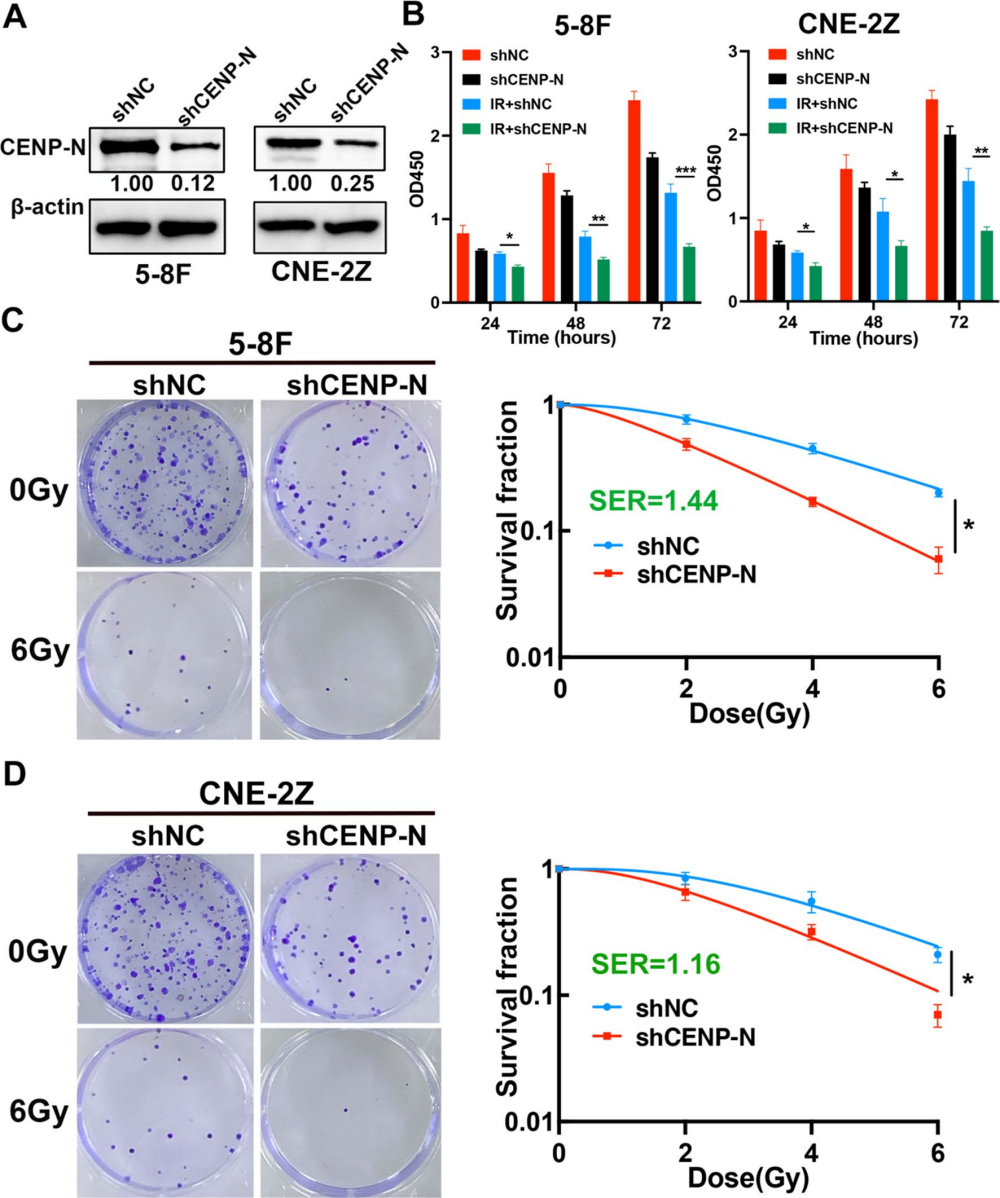
Knockdown of CENP-N inhibits NPC cell proliferation and enhances radiosensitivity. **A** Western blotting was used to validate the expression of CENP-N in 5-8 F and CNE-2Z cells. **B** Cell proliferation viability assessed by the CCK-8 assay after knockdown of CENP-N and/or radiation treatment. **C, D** Colony formation assay measuring the colony-forming ability of cells following knockdown of CENP-N and/or radiation treatment. *p < 0.05, **p < 0.01, ***p < 0.001

Firstly, we used shCENP-N plasmids and packaging plasmids (pMD2G and psPAX2) to transfect 293T. After 48 h, collecting the lentivirus-containing supernatant to infect NPC cells. Then purinomycin was used to kill cells that were not successfully transfected. Compared to shNC group, the CENP-N expression was significantly reduced by more than 70% in the shCENP-N group (Fig. 2A). The proliferation activity of cells treated with radiotherapy (a single dose of 6 Gy at 400 cGy/min for 1 min) or CENP-N knockdown or both was assessed using CCK8. The results showed that IR + shCENP-N group exhibited the most significant suppression of cell proliferation after 24, 48, or 72 h (Fig. 2B, IR + shCENP-N vs. shCENP-N or IR + shNC). In addition, the IR + shCENP-N group exhibited a noteworthy decrease in the number of cells when compared to the IR + shNC group (Fig. 2C, D). According to the analysis of radiosensitivity parameters (Table 2), when the radiation dose was 2 Gy, the cell survival fractions in the control group were 0.81 (5-8 F) and 0.91 (CNE-2Z), whereas the fractions were 0.52 (5-8 F) and 0.72 (CNE-2Z) after CENP-N knockdown, and the SER were 1.44 (5-8 F) and 1.16 (CNE-2Z), respectively.

**Table 2:** Radiobiological parameters fit to the single-hit multitarget model

Cell line	Groups	D_0_(Gy)	D_q_(Gy)	N	SF2	k	SER
5-8 F	IR + shNC	2.56	2.88	3.08	0.81	0.39	1.44
	IR + shCENP-N	1.79	1.24	2.00	0.52	0.56	
CNE-2Z	IR + shNC	2.22	3.04	3.92	0.91	0.45	1.16
	IR + shCENP-N	1.92	1.78	2.52	0.72	0.52	

 D_0_ , average lethal dose; D_q_, quasi-threshold dose; SF2, survival fraction (2 Gy); SER, sensitization enhancement ratio.

### Knockdown of CENP-N enhances radiation-induced G2/M phase arrest and cell apoptosis

Flow cytometry was utilized to examine the cell cycle distribution and assess apoptosis.

**Fig. 3:**
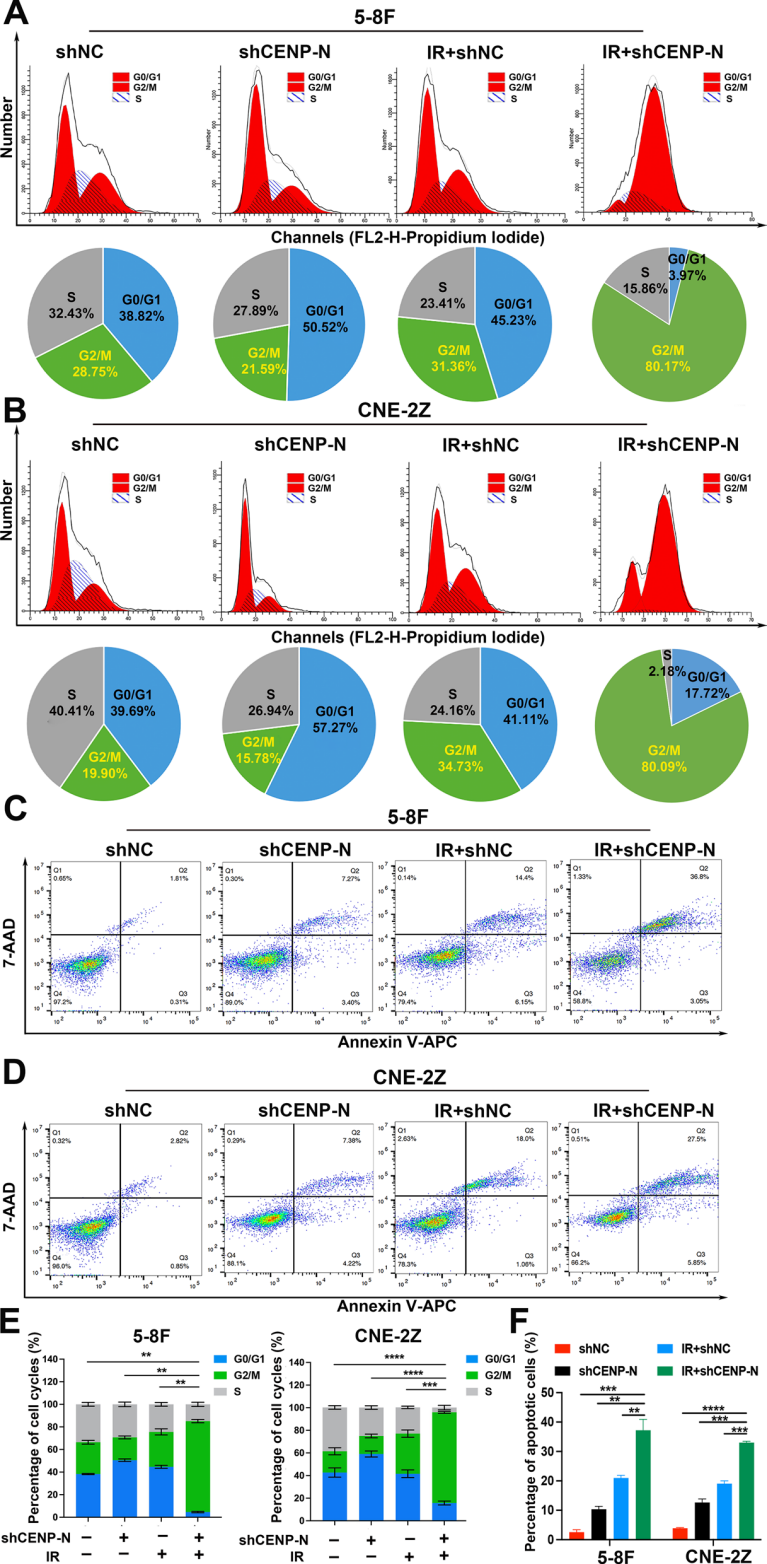
Knockdown of CENP-N enhances radiation-induced G2/M phase arrest and cell apoptosis. **A, B** Flow cytometry determined the distribution of cells in various stages of the cell cycle and quantitatively analyzed 5-8 F and CNE-2Z cells treated with CENP-N knockdown and/or radiation. **C, D** Flow cytometry detected apoptotic cells and quantitatively analyzed 5-8 F and CNE-2Z cells treated with CENP-N knockdown and/or radiation. **E, F** Statistical analysis of changes in the distribution of cells in various stages of the cell cycle and apoptotic cells among NPC cells after combined treatment with CENP-N knockdown and radiation. **p < 0.01, ***p < 0.001, ****p < 0.0001

In the shCENP-N and IR + shNC groups, the proportions of radiation-sensitive G2/M phase cells among 5-8 F cells were 22.30 ± 1.00% and 29.68 ± 2.38%, respectively. However, in the IR + shCENP-N group, the proportion of radiation-sensitive G2/M phase cells among 5-8 F cells increased to 84.96 ± 6.77% (Fig. 3A, E). Similarly, in the shCENP-N or IR + shNC group, the proportions of radiation-sensitive G2/M phase cells among CNE-2Z cells was 15.08 ± 0.99% and 33.73 ± 1.42%, respectively. However, in the IR + shCENP-N group, the proportion of radiation-sensitive G2/M phase cells among CNE-2Z cells increased to 78.73 ± 1.93% (Fig. 3B, E).

In the shCENP-N and IR + shNC groups, the apoptosis rates of 5-8 F cells were 10.34 ± 1.02% and 20.97 ± 0.92%, respectively. However, in the IR + shCENP-N group, the apoptosis rate of 5-8 F cells increased to 37.23 ± 3.71% (Fig. 3C, F). Similarly, in the shCENP-N and IR + shNC groups, the apoptosis rates of CNE-2Z cells were 12.66 ± 1.19% and 19.07 ± 0.96%, respectively. However, when CENP-N knockdown was combined with radiation treatment, the apoptosis rate of CNE-2Z cells increased to 33.03 ± 0.46% (Fig. 3D, F).

These results collectively validate that knockdown of CENP-N can enhance radiation-induced G2/M phase arrest and cell apoptosis.

### Knockdown of CENP-N affects the AKT/mTOR signaling pathway, cell apoptosis, DNA damage and cell cycle-related protein expression in NPC cells

Previous studies have demonstrated a correlation between AKT/mTOR and the susceptibility of cancer cells to radiation [11]. In order to investigate the possible mechanism by which knocking down CENP-N improves the radiosensitivity of NPC cells, the cells were exposed to a single dose of 6 Gy at 400 cGy/min for 1 min, after 24 h, the cellular proteins were extracted to detect the effect of knocking down CENP-N combined with radiotherapy on this signaling pathway and the expression of proteins associated with cell apoptosis, DNA damage, and cell cycle.

**Fig. 4:**
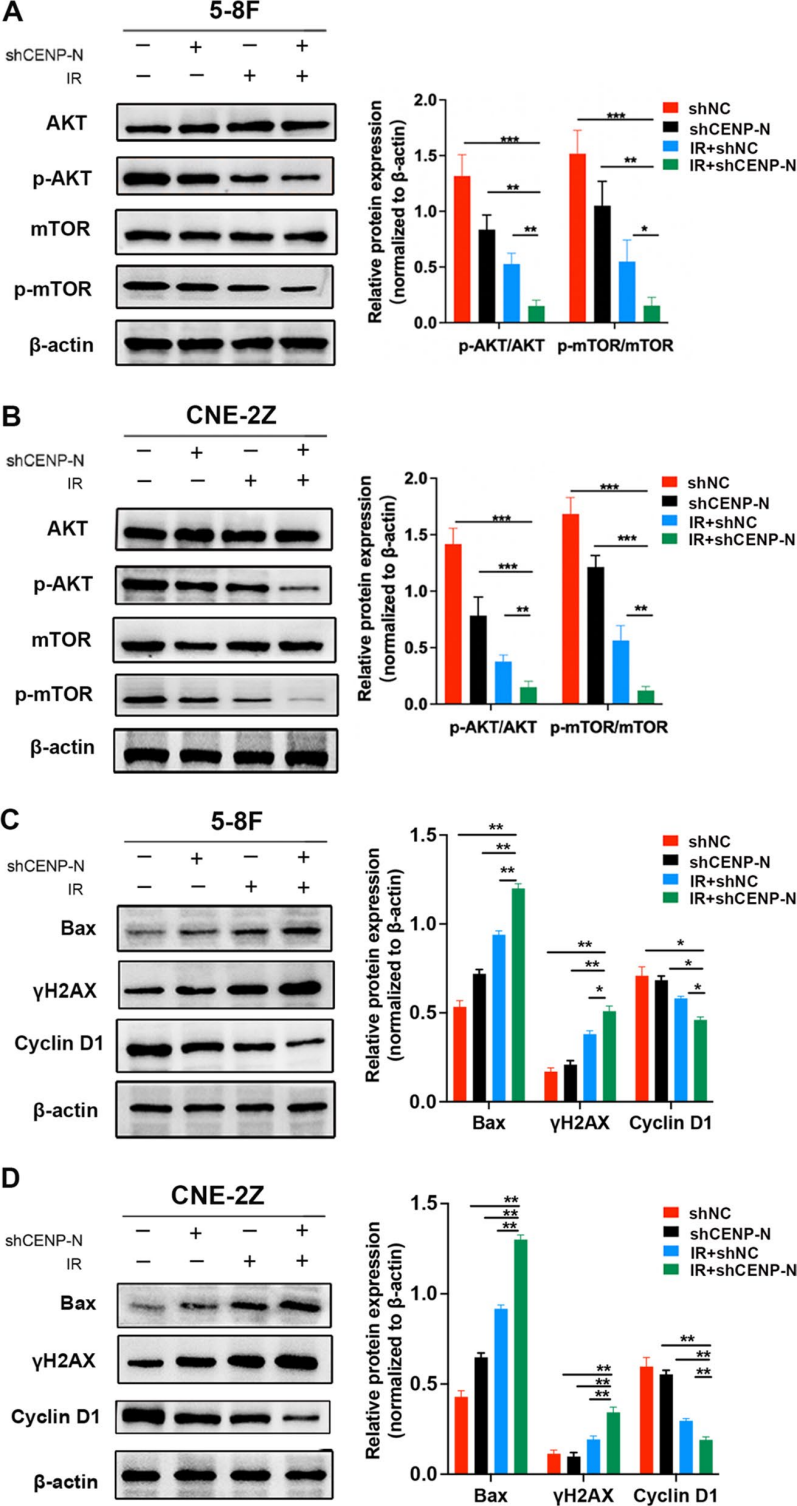
Knockdown of CENP-N affects the expression of proteins related to the AKT/mTOR signaling pathway, cell apoptosis, DNA damage, and the cell cycle in NPC cells. **A, B** Western blot detected AKT/mTOR pathway-related proteins expression in cells treated with CENP-N knockdown and/or radiotherapy. **C, D** Western blot detected Bax, γH2AX, and Cyclin D1 expression following CENP-N knockdown and/or radiotherapy. *p < 0.05, **p < 0.01, ***p < 0.001

In 5-8 F and CNE-2Z cells, the expression of AKT/mTOR pathway-related proteins was significantly lower in IR + shCENP-N group than in IR + shNC or shCENP-N group(Fig. 4A, B); the levels of the apoptosis-related protein Bax and DNA damage marker protein γH2AX in IR + shCENP-N group were significantly higher than that in the other three groups. Conversely, the expression of the cell cycle regulatory protein Cyclin D1 was significantly lower than that in the other three groups (Fig. 4C, D). These results confirmed that knockdown of CENP-N combined with radiation therapy may increase radiation-induced apoptosis, DNA damage, and G2/M phase arrest by inhibiting AKT/mTOR pathway, thereby enhancing the NPC radiosensitivity.

### Knockdown of CENP-N combined with radiotherapy suppresses the proliferation of NPC cells through the AKT/mTOR signaling pathway

**Fig. 5:**
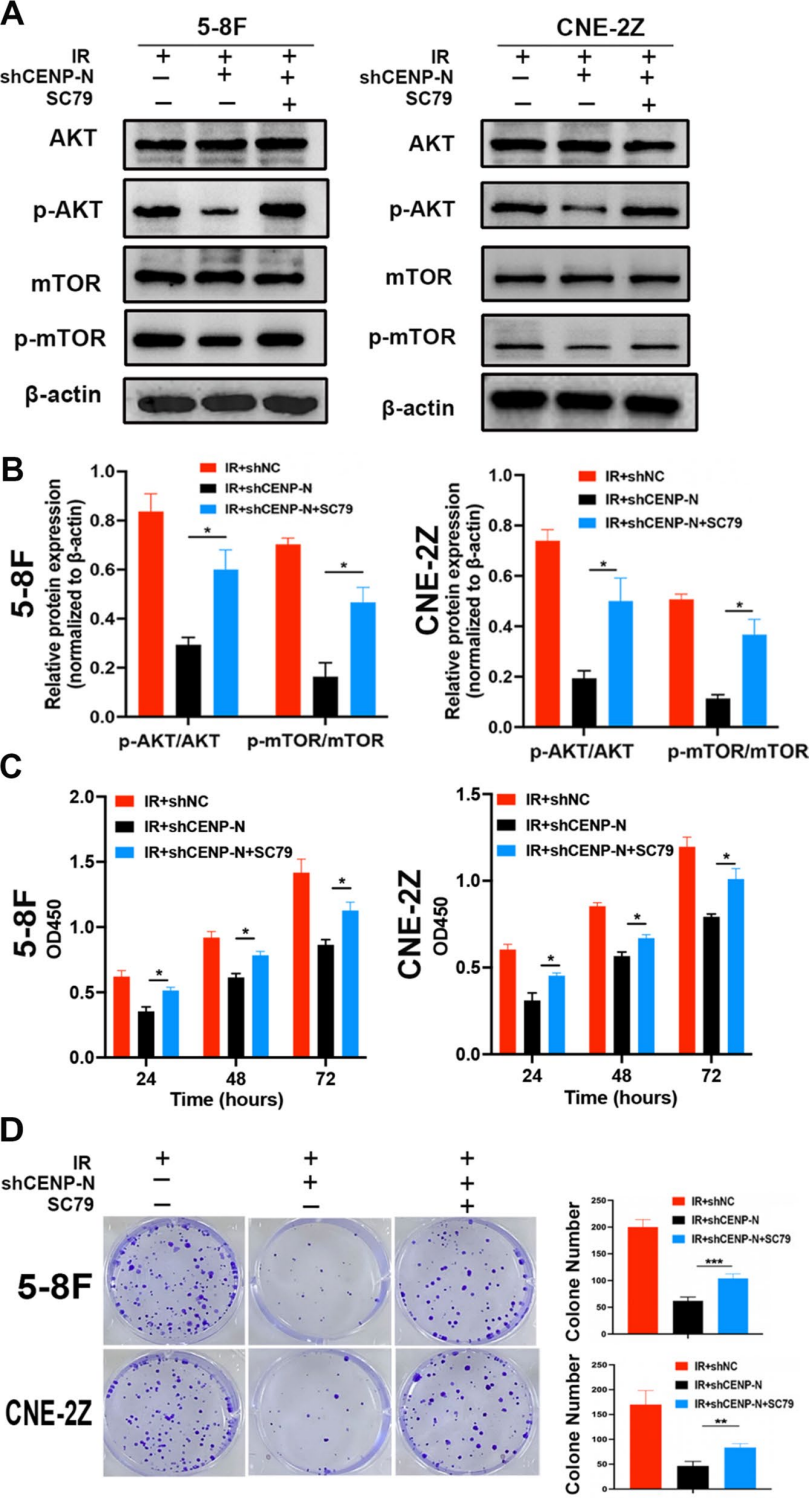
Knockdown of CENP-N combined with radiotherapy suppresses the proliferation of NPC cells through the AKT/mTOR signaling pathway. **A. B** Western blot detected AKT/mTOR signaling pathway-related proteins expression. **C** CCK-8 assay detected the proliferation of NPC cells treated with CENP-N knockdown and/or SC79 (5 µg/mL) after radiotherapy. **D** Colony formation assay evaluating the colony-forming ability of NPC cells treated with CENP-N knockdown and/or SC79 (5 µg/mL) after radiotherapy. *p < 0.05, **p < 0.01, ***p < 0.001

To further validate whether knockdown of CENP-N combined with radiotherapy inhibits the proliferation of NPC cells by suppressing the AKT/mTOR signaling pathway, we treated CENP-N knockdown NPC cells with the AKT activator SC79 (5 ug/mL) to activate the AKT/mTOR signaling pathway (Fig. 5A, B). Compared to IR + shCENP-N group, the proliferation rate in IR + shCENP-N + SC79 group significantly increased (Fig. 5C). Consistent with these findings, the results of the colony formation assay indicated a notable reduction in the number of NPC cell colonies in the CENP-N knockdown and radiation combined group, while treatment with the AKT activator SC79 led to an increase in the number of cell colonies (Fig. 5D). These results strongly confirmed that CENP-N combined with radiotherapy suppresses the proliferation of NPC cells after radiation by suppressing the AKT/mTOR signaling pathway.

### Knockdown of CENP-N enhances radiation-induced apoptosis, DNA damage, and G2/M phase arrest through the AKT/mTOR signaling pathway

Subsequently, we assessed apoptosis and the levels of DNA damage- and cell cycle distribution-related proteins in NPC cells after treatment with the AKT activator SC79 following CENP-N knockdown.

**Fig. 6:**
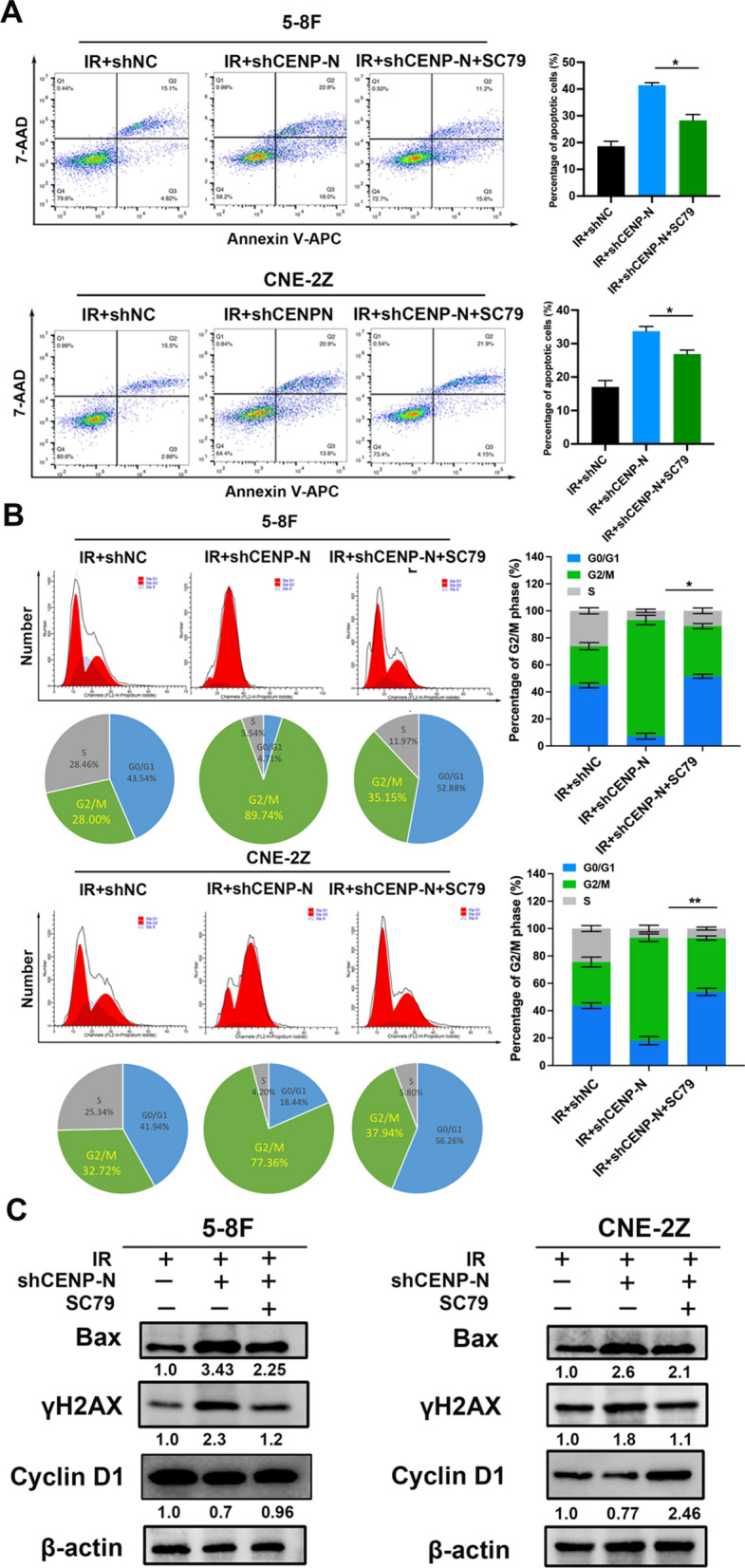
Knockdown of CENP-N regulates cell apoptosis and cell cycle distribution in NPC cells by inhibiting the AKT/mTOR signaling pathway. **A** Flow cytometry analysis of the percentage of apoptotic NPC cells following irradiation with CENP-N knockdown and SC79 treatment. **B** Flow cytometry analysis of the G2/M phase cell percentage among NPC cells following radiation with CENP-N knockdown and SC79 treatment. **C** Western blot analysis of the protein expression levels of Bax, γH2AX and Cyclin D1 in NPC cells following radiation with CENP-N knockdown and SC79 treatment. *p < 0.05, **p < 0.01

In IR + shNC groups, the apoptosis rates were 18.60 ± 1.87% (5-8 F) and 17.04 ± 1.90% (CNE-2Z). In the IR + shCENP-N group, the apoptosis rates increased to 41.45 ± 0.92% (5-8 F) and 33.70 ± 1.41% (CNE-2Z). However, in IR + shCENP-N + SC79 group, the apoptosis rates were 28.33 ± 2.16% (5-8 F) and 26.88 ± 1.17% (CNE-2Z). Overall, compared with the radiation-alone group, a significant increase in apoptosis was observed in 5-8 F and CNE-2Z cells after CENP-N knockdown combined with radiation, which was reversed upon treatment with the AKT activator SC79 (Fig. 6A). Consistently, the expression of the apoptosis-related protein Bax was elevated after CENP-N knockdown combined with radiation but decreased again after treatment with SC79 (Fig. 6C).

In IR + shNC groups, the percentages of G2/M phase were 29.68 ± 2.38% (5-8 F) and 33.73 ± 1.42% (CNE-2Z). In the IR + shCENP-N group, the percentages of G2/M phase increased to 84.96 ± 6.77% (5-8 F) and 78.73 ± 1.93% (CNE-2Z). However, in IR + shCENP-N + SC79 group, the percentages of G2/M phase were 31.88 ± 4.63% (5-8 F) and 34.12 ± 5.40% (CNE-2Z). Overall, relative to radiation alone, the proportion of G2/M phase cells, which are more sensitive to radiation, significantly increased among 5-8 F and CNE-2Z cells after CENP-N knockdown combined with radiation, and this phenomenon was reversed upon treatment with SC79 (Fig. 6B). Similarly, the expression of the cell cycle regulatory protein Cyclin D1 decreased after CENP-N knockdown but increased after SC79 treatment (Fig. 6C). Additionally, compared to radiation alone, the expression of the DNA damage marker protein γH2AX was upregulated after CENP-N knockdown combined with radiation but decreased after SC79 treatment (Fig. 6C, B).

These results confirmed that CENP-N knockdown enhances radiation-induced apoptosis, DNA damage, and G2/M phase arrest in NPC cells through the inhibition of the AKT/mTOR signaling pathway.

### Knockdown of CENP-N enhances the radiosensitivity of NPC in vivo

**Fig. 7:**
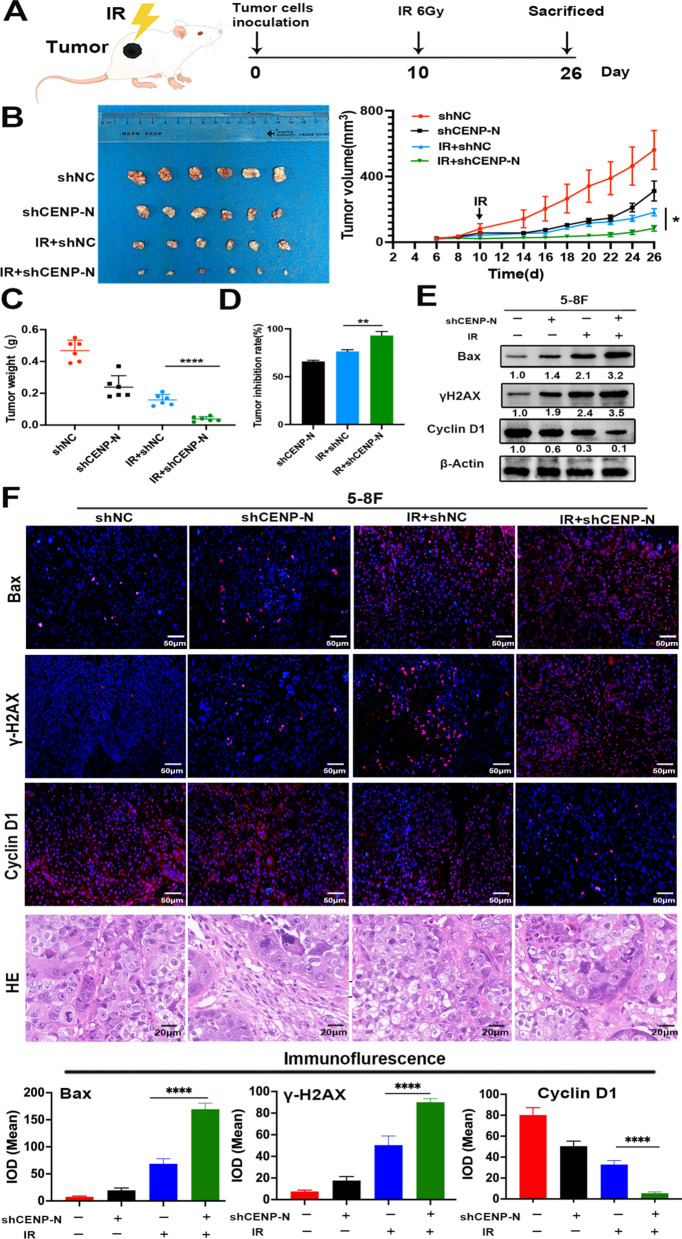
Knockdown of CENP-N enhances the radiosensitivity of NPC in vivo. **A** Subcutaneous xenograft model of NPC in nude mice. **B** Tumor images and growth curves of nude mice treated with CENP-N knockdown, radiotherapy, or both. **C** Average tumor weight in nude mice treated with CENP-N knockdown, radiotherapy, or both. **D** Tumor inhibition rate after CENP-N knockdown, radiotherapy, or both. **E** Western blot analysis of the protein expression levels of Bax, γH2AX and Cyclin D1 in tumor tissues treated with CENP-N knockdown, radiotherapy, or both. **F** Representative images of tumor tissues stained with immunofluorescence and H&E after CENP-N knockdown, radiotherapy, or both. *p < 0.05, **p < 0.01, ****p < 0.0001

Next, we established a subcutaneous xenograft model of NPC in nude mice (Fig. 7A). By measuring tumor growth in nude mice in vivo, it was found that the average tumor volume and weight in the IR + shNC group were (182 ± 54) mm^3^ and (0.16 ± 0.03) g, respectively, whereas in the IR + shCENP-N group, these values were significantly reduced to (84 ± 42) mm^3^ and (0.04 ± 0.01) g, respectively, with a statistically significant difference (Fig. 7B, C). Moreover, the tumor inhibition rate in the IR + shCENP-N group was 93.0%, which was significantly higher than the rates of 76% in the IR + shNC group and 66% in the shCENP-N group (Fig. 7D).

In addition, Western blot and immunofluorescence were used to determine the protein expression changes in Bax, γH2AX and Cyclin D1 within the transplanted tumor tissues of nude mice. Our findings showed that the expression levels of Bax and γH2AX were significantly higher in the CENP-N knockdown combined with radiotherapy group than in the radiotherapy alone group; the expression level of Cyclin D1 was lower in the CENP-N knockdown combined with radiotherapy group than in the radiotherapy alone group (Fig. 7E, F). The findings were in line with the in vitro experimental outcomes.

## Discussion

NPC is a highly malignant epithelial tumor. Due to its unique anatomical location and proximity to vital organs, radical surgical resection is challenging [2]. Radiotherapy is currently the recommended therapy for NPC patients. However, during the course of radiotherapy, many gene and protein levels are altered, leading to decreased sensitivity of tumor cells to radiation and resulting in local recurrence and distant metastasis in some patients [4]. Clinical trials are currently testing various chemotherapeutic medicines, such as 5-fluorouracil and platinum analogs, to enhance the effectiveness of radiation therapy. Although the general prognosis has improved, some patients still relapse following treatment, and the prognosis is dismal [28]. DNA damage repair, cell cycle arrest, autophagy and apoptosis, tumor stemness, and immunological response have all been found to be involved in the regulation of radiation resistance. Ionizing radiation either directly acts on target cell DNA or acts on water to generate free radicals, causing target cell DNA damage [29]. In eukaryotes, DNA damage can be repaired via the DNA damage response that follows radiation, and aberrant DNA damage repair can have a significant impact on tumor cell survival and lead to radioresistance [30]. Therefore, exploring the molecular mechanisms underlying radiosensitization in NPC, identifying novel radiotherapy targets, and improving the prognosis of NPC patients are of significant importance.

Akt/mTOR activation has been shown to enhance radioresistance in many kinds of tumors, including HNSCC, esophageal cancer, colorectal cancer, lung cancer, and brain cancer [31–34]. Clinical investigations have also described the predictive value of activated Akt in terms of predicting response to radiation in solid malignancies. The results of immunohistochemical analysis of Akt S473 phosphorylation indicate that activated Akt is a potential predictive biomarker of radiotherapy response in patients with HNSCC and cervical cancer [35, 36]. By suppressing the AKT/mTOR signaling pathway, exosomes loaded with miR-197-3p augment the sensitivity of NPC to radiochemotherapy [37]. Xie et al. found that overexpression of C2orf40 can suppress the activity of the AKT/mTOR signaling pathway and enhance the sensitivity of NPC cells to radiochemotherapy [6]. As a result, combining radiotherapy and molecularly targeted therapy may be one of the most effective cancer treatment techniques. In this study, we confirmed that CENP-N knockdown inhibited AKT/mTOR pathway, and enhanced NPC radiosensitivity. However, this effect was reversed when the AKT activator SC79 was used. Thus, our study demonstrated that downregulation of CENP-N expression enhances the radiosensitivity of NPC cells by downregulating the expression of proteins in AKT/mTOR pathway.

CENP-N binds to CENP-A nucleosomes in a DNA sequence-independent manner in the early stages of cell mitosis to maintain the stability of kinetochores and microtubule connections [38]. CENP-N mutations cause metaphase kinetochore assembly and mitotic defects [39]. Studies have found that both metabolic pathways, glycolysis and oxidative phosphorylation, can regulate tumor radiotherapy sensitivity [40]. Previously, we have found that NPC patients with low expression of CENP-N have decreased glucose uptake and inhibited glycolysis in primary tumor tissues [41]. Therefore, CENP-N may also regulate the radiosensitivity of NPC cells by regulating the glycolysis level. The nucleotide synthesis pathway is also involved in the radiotherapy resistance of pancreatic cancer [42]. For example, upregulation of glutamine synthetase can promote nucleotide biosynthesis and promote DNA repair in nasopharyngeal cancer cells, thereby enhancing their radiotherapy resistance [43]. In future studies, we can further investigate CENP-N’s effects on various metabolic pathways and explore whether CENP-N regulates the radiosensitivity of NPC through metabolic reprogramming.

Bax, an important regulator of apoptosis. Decreased Bax expression leads to resistance to cell apoptosis, while increased Bax expression enhances the sensitivity of tumor cells to apoptosis [44]. Wang et al. found that patients with higher levels of Bax protein expression in 96 glioma samples had significantly improved survival rates after radiotherapy [45]. During our investigation, we noticed a notable increase in the rate of cell apoptosis and Bax expression after CENP-N knockdown combined with radiotherapy, while the expression level of Bax decreased following treatment with the AKT activator SC79. These findings confirmed that CENP-N, through the regulation of the AKT/mTOR signaling pathway, upregulates Bax expression, leading to increased cell apoptosis after radiotherapy.

The γH2AX protein is synthesized in response to the occurrence of DNA double-strand breaks. Its localization is consistently determined and directly correlates with the quantity of broken DNA strands in a 1:1 manner. The utilization of this particular quantitative evaluation technique is prevalent in assessing the biological impacts of X-ray radiation [46]. The upregulation of AKR1B10 has the potential to stimulate the creation of free fatty acids (FFAs) in NPC. This, in turn, activates TLR4/NF-ĸB pathway, leading to a decrease in the levels of γH2AX. Consequently, this molecular cascade enhances the resistance of NPC to radiotherapy [47]. In this study, we observed a significant increase in the protein level of γH2AX after CENP-N knockdown combined with radiotherapy, indicating DNA damage. However, treatment with the AKT activator SC79 induced a reduction in γH2AX protein. The findings validate that suppression of CENP-N can impede the repair of DNA damage by means of the AKT/mTOR signaling pathway, consequently amplifying the extent of double-strand DNA damage caused by radiation.

Several studies have shown that actively dividing tumor cells are more susceptible to radiation damage. Tumor cells in the G2/M phase exhibit more active DNA separation and higher cell division activity, making them more vulnerable to the DNA and cell damage caused by radiation, ultimately leading to programmed cell death [48]. Chang et al. found that siRNA-mediated suppression of GP96 and GDF-15 expression in radiation-resistant NPC cells resulted in G2/M cell cycle arrest, inhibited proliferation, and impaired colony formation [49]. The expression level of Cyclin D1, which is highly sensitive to proliferative signals from growth factor receptors, Ras, and downstream effector molecules, is critical for the regulation of G1 phase, initiation of DNA synthesis and subsequent inhibition in the S phase to ensure efficient DNA synthesis [50]. Raj et al. found that the drug BRM270 induced G2/M phase arrest, effectively inhibited proliferation, and induced apoptosis in hepatocellular carcinoma through downregulation of the Cyclin D1/Bcl2-mediated c-Jun apoptosis pathway [51]. The cell cycle is directly regulated by p16. By inhibiting CDK4, transcribing E2F factors, and maintaining Rb protein dephosphorylation, it can stop the cell cycle in G1. p16 is inactivated, the cell cycle is shortened, and the cell cycle reaches the S phase early, reducing NPC radiosensitivity [52]. In our study, we found that CENP-N knockdown alone leads to G0/G1 phase arrest, but CENP-N knockdown combined with radiotherapy leads to G2/M phase arrest, accompanied by a significant decrease in Cyclin D1 expression, indicating that the effect of CENP-N on phenotype was mainly achieved by radiation-induced G2/M phase arrest. However, treatment with the AKT activator SC79 resulted in a notable recovery of Cyclin D1 expression. Therefore, the knockdown of CENP-N combined with radiotherapy enhances radiation damage by suppressing the AKT/mTOR signaling pathway, downregulating Cyclin D1, and sensitizing NPC cells to radiotherapy during the G2/M phase.

He et al. reported that ApoG2 can induce cell apoptosis by disrupting the binding of Bcl-2 and Bax, arresting the cell cycle in the S phase, and consequently enhancing the radiosensitivity of CNE-2 NPC xenografts in nude mice, resulting in a tumor suppression rate of 61.64% [53]. In our study, the tumor suppression rate of the combined knockdown of CENP-N and radiotherapy group was 93.0%. Nie et al. found that the sensitization ratio of NPC cells after knocking down SALL4 was 1.11 [54], while Zhang et al. reported a sensitization ratio of 1.21 after knocking down RPA1 [55]. In our study, we observed a sensitization ratio of 1.44 after knocking down CENP-N. Therefore, compared to other reported genes, CENP-N has more advantages in improving NPC radiosensitivity.

In recent years, tumor hyperthermia has been considered as a green and non-toxic radiosensitizing technique. The sensitization mechanisms include improving the degree of tumor hypoxia and inhibiting DNA damage repair [56]. Nanomaterials have also received extensive attention in radiotherapy due to their good biocompatibility and safety [57, 58]. Therefore, many researchers combine the two methods and use nano-hyperthermia technology as a sensitizing carrier to achieve radiosensitization [59]. Some characteristic nanoparticles have strong X-ray absorption ability, which can greatly improve the X-ray absorption in the deep part of the tumor tissue and further improve the effect of tumor radiotherapy [60]. In addition, functionalized carbon nanotubes (CNTS) can be used as nanocarriers in gene therapy to deliver biological small molecules such as microRNA or shRNA [61]. In the future, we may be able to deliver shRNA or small molecule drugs targeting CENP-N via nanomaterials to enhance the radiosensitivity of NPC. In vivo studies may be conducted on different animal models to explore whether other signal pathways are involved in CENP-N-mediated radioresistance, and whether there are potential side effects or other challenges. Subsequently, animal experiments will be transferred to clinical trials.

By analyzing 35 NPC tissue samples, it was observed that the expression of CENP-N was notably reduced in the radiosensitive tissues in comparison to the radioresistant tissues. When CENP-N was knocked down more than 70% in vitro, the sensitization ratio of 5-8 F cells was 1.44 (5-8 F), and the sensitization ratio of CNE-2Z cells was 1.16. In vivo studies further confirmed that CENP-N knockdown combined with radiotherapy had a tumor inhibition rate of up to 93%.

However, this study also has several limitations. First, we selected a relatively small number of clinical samples, and these results need to be further validated in a larger and more diverse patient population. Second, only two NPC cell lines were used to verify the radiosensitizing effect of CENP-N knockdown in vitro. More NPC cell lines may be used to comprehensively verify the effect of CENP-N on radiosensitivity of NPC cells. Thirdly, mechanistically, we focused on the effect of CENP-N on AKT/mTOR signaling pathway, which, although important, may not be the only pathway regulating tumor radiosensitivity. Therefore, we will extensively investigate the effects of CENP-N on other pathways in the future, hoping to lay the foundation for the use of nanomaterials to deliver CENP-N-targeting shRNA or other small molecule drugs for NPC radiotherapy.

Taken together, our study demonstrated that CENP-N knockdown enhanced the radiosensitivity of NPC by inhibiting AKT/mTOR pathway.

### Supplementary Information

**Additional file 1: Figure S1.** CENP-N was significantly reduced in radiosensitive NPC. **A** Representative immunohistochemical images of CENP-N, p-AKT, and p-mTOR in radiotherapy-sensitive and radiotherapy-resistant tissue samples. **B** Immunohisto- chemical scores of CENP-N, p-AKT, and p-mTOR in 35 NPC samples. **C** The expression of CENP-N in HNSCC and normal tissues. *p<0.05,**p<0.01,. ****p<0.0001.

**Additional file 2: Figure S2.** Flow cytometry gating representative plots. **A** Representative cell cycle flow cytometry gating representative plots. **B** Representative cell apoptosis flow cytometry gating representative plots. **Table S1.** The information for all antibodies used. **Table S2.** The information of percentages for cell cycle phase. **Table S3.** The information of percentages for apoptosis rate.

**Additional file 3: Table S1.**  The information for all antibodies used. **Table S2.** The information of percentages for cell cycle phase. **Table S3.** The information of percentages for apoptosis rate.

## Data Availability

All data generated or analyzed during this study are included in this published article and its supplementary information files.
