# Supplementary figures and images for: Enhancing nasopharyngeal carcinoma cell radiosensitivity by suppressing AKT/mTOR via CENP-N knockdown
Source: J Transl Med. 2023 Nov 8;21:792. doi: 10.1186/s12967-023-04654-x (PMC10631041; doi:10.1186/s12967-023-04654-x)

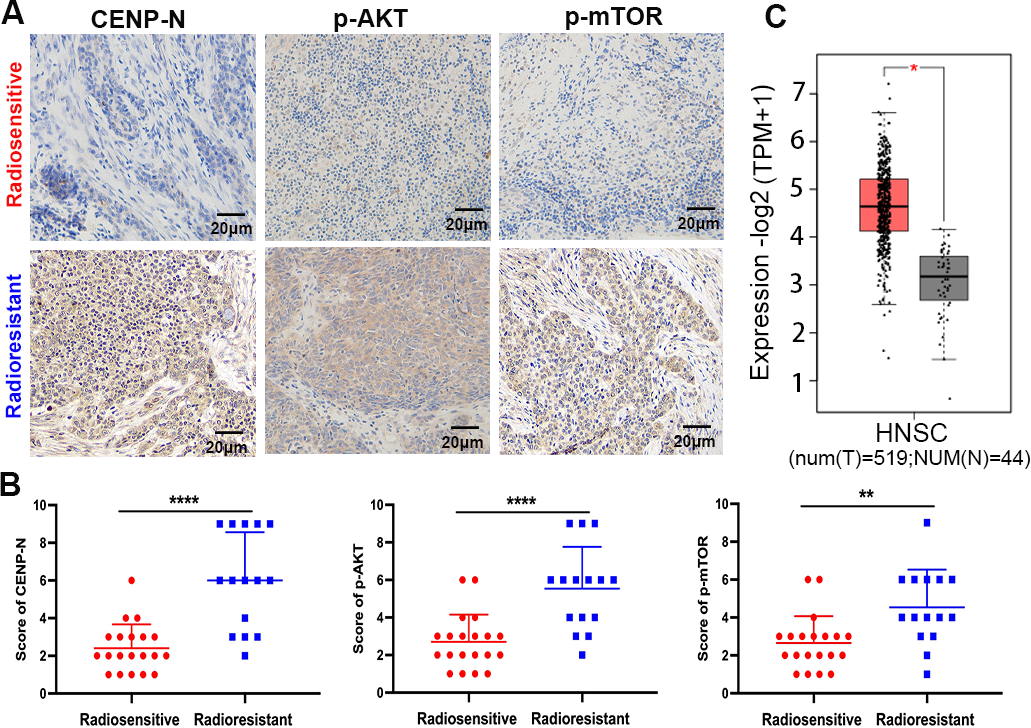

Supplement: Supplementary file 1 — Additional file 1: Figure S1. CENP-N was significantly reduced in radiosensitive NPC. A Representative immunohistochemical images of CENP-N, p-AKT, and p-mTOR in radiotherapy-sensitive and radiotherapy-resistant tissue samples. B Immunohisto- chemical scores of CENP-N, p-AKT, and p-mTOR in 35 NPC samples. C The expression of CENP-N in HNSCC and normal tissues. *p<0.05,**p<0.01,. ****p<0.0001. [file 12967_2023_4654_MOESM1_ESM.png]

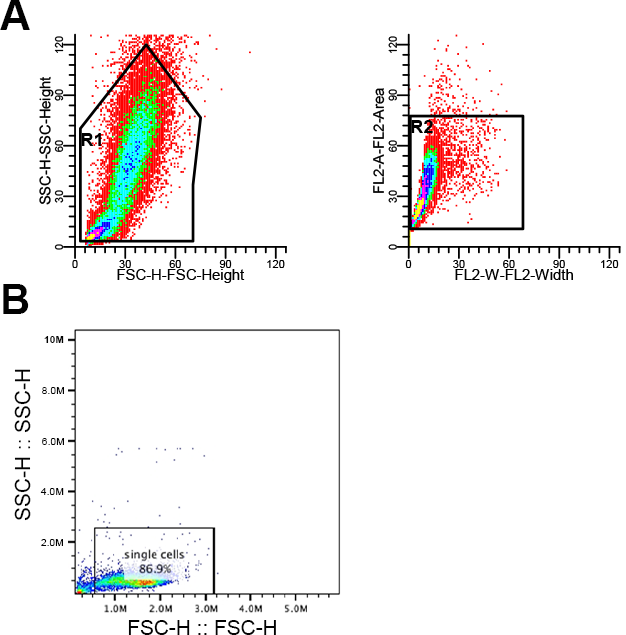

Supplement: Supplementary file 2 — Additional file 2: Figure S2. Flow cytometry gating representative plots. A Representative cell cycle flow cytometry gating representative plots. B Representative cell apoptosis flow cytometry gating representative plots. Table S1. The information for all antibodies used. Table S2. The information of percentages for cell cycle phase. Table S3. The information of percentages for apoptosis rate. [file 12967_2023_4654_MOESM2_ESM.png]
