# Supplementary material for: Enhancing nasopharyngeal carcinoma cell radiosensitivity by suppressing AKT/mTOR via CENP-N knockdown
Source: J Transl Med. 2023 Nov 8;21:792. doi: 10.1186/s12967-023-04654-x (PMC10631041; doi:10.1186/s12967-023-04654-x)
Supplement: Supplementary file 3 — Additional file 3: Table S1. The information for all antibodies used. Table S2. The information of percentages for cell cycle phase. Table S3. The information of percentages for apoptosis rate. [file 12967_2023_4654_MOESM3_ESM.docx]

**Additional Table 1 The information for all antibodies used**

| **Antibodies** | **Company** | **Number** | **Country** | **Applications** |
| --- | --- | --- | --- | --- |
| CENP-N | Affinity | DF2315 | USA | WB,IHC,IF |
| p-AKT | Affinity | AF0016 | USA | WB,IHC,IF |
| p-mTOR | Affinity | AF3308 | USA | WB,IHC,IF |
| Bax | Affinity | AF0120 | USA | WB,IHC,IF |
| γH2AX | Affinity | AF3187 | USA | WB,IHC,IF |
| Cyclin D1 | Affinity | AF0931 | USA | WB,IHC,IF |
| Secondary Antibody | Servicebio | G1213 | China | WB,IHC |
| CY3-labeled goat anti-rabbit fluorescent secondary antibody | Servicebio | GB21303 | China | IF |

**Additional Table 2** The information of percentages for cell cycle phase.

| Cell lines | Groups | G0/G1 | S | G2/M |
| --- | --- | --- | --- | --- |
| 5-8F | shNC | 38.82% | 32.43% | 28.75% |
|  | shCENP-N | 50.52% | 27.89% | 21.59% |
|  | IR+shNC | 45.23% | 23.41% | 31.36% |
|  | IR+shCENP-N | 3.97% | 15.86% | 80.17% |
| CNE-2Z | shNC | 39.69% | 40.41% | 19.90% |
|  | shCENP-N | 57.27% | 26.94% | 15.78% |
|  | IR+shNC | 41.11% | 24.16% | 34.73% |
|  | IR+shCENP-N | 17.72% | 2.18% | 80.09% |

**Additional Table 3** The information of percentages for apoptosis rate.

| Cell lines | Groups | APC(-) | APC(+) | APC(+) | APC(-) |
| --- | --- | --- | --- | --- | --- |
|  |  | 7-AAD(+) | 7-AAD(+) | 7-AAD(-) | 7-AAD(-) |
| 5-8F | shNC | 0.65% | 1.81% | 0.31% | 97.20% |
|  | shCENP-N | 0.30% | 7.27% | 3.40% | 89.00% |
|  | IR+shNC | 0.14% | 14.40% | 6.15% | 79.40% |
|  | IR+shCENP-N | 1.33% | 36.80% | 3.05% | 58.80% |
| CNE-2Z | shNC | 0.32% | 2.82% | 0.85% | 96.00% |
|  | shCENP-N | 0.29% | 7.38% | 4.22% | 88.10% |
|  | IR+shNC | 2.63% | 18.00% | 1.06% | 78.30% |
|  | IR+shCENP-N | 0.51% | 27.50% | 5.85% | 66.20% |
